# Next-generation sequencing for diagnosis of thoracic aortic aneurysms and dissections: diagnostic yield, novel mutations and genotype phenotype correlations

**DOI:** 10.1186/s12967-016-0870-4

**Published:** 2016-05-04

**Authors:** J. K. Poninska, Z. T. Bilinska, M. Franaszczyk, E. Michalak, M. Rydzanicz, E. Szpakowski, A. Pollak, B. Milanowska, G. Truszkowska, P. Chmielewski, A. Sioma, H. Janaszek-Sitkowska, A. Klisiewicz, I. Michalowska, M. Makowiecka-Ciesla, P. Kolsut, P. Stawinski, B. Foss-Nieradko, M. Szperl, J. Grzybowski, P. Hoffman, A. Januszewicz, M. Kusmierczyk, R. Ploski

**Affiliations:** Molecular Biology Laboratory, Institute of Cardiology, Warsaw, Poland; Unit for Screening Studies in Inherited Cardiovascular Diseases, Institute of Cardiology, Warsaw, Poland; Department of Medical Genetics, Centre of Biostructure, Medical University of Warsaw, Warsaw, Poland; Department of Cardiac Surgery and Transplantation, Institute of Cardiology, Warsaw, Poland; Department of Genetics, Institute of Physiology and Pathology of Hearing, Warsaw, Poland; Department of Hypertension, Institute of Cardiology, Warsaw, Poland; Department of Congenital Cardiac Defects, Institute of Cardiology, Warsaw, Poland; Department of Radiology, Institute of Cardiology, Warsaw, Poland; Department of Cardiomyopathy, Institute of Cardiology, Warsaw, Poland

**Keywords:** Thoracic aortic aneurysm and dissections, Next-generation sequencing, Diagnosis, Marfan syndrome, Loeys–Dietz syndrome, Shprintzen–Goldberg syndrome

## Abstract

**Background:**

Thoracic aortic aneurysms and dissections (TAAD) are silent but possibly lethal condition with up to 40 % of cases being hereditary. Genetic background is heterogeneous. Recently next-generation sequencing enabled efficient and cost-effective examination of gene panels. Aim of the study was to define the diagnostic yield of NGS in the 51 TAAD patients and to look for genotype–phenotype correlations within families of the patients with TAAD.

**Methods:**

51 unrelated TAAD patients were examined by either whole exome sequencing or TruSight One sequencing panel. We analyzed rare variants in 10 established thoracic aortic aneurysms-associated genes. Whenever possible, we looked for co-segregation in the families. Kaplan–Meier survival curve was constructed to compare the event-free survival depending on genotype. Aortic events were defined as acute aortic dissection or first planned aortic surgery.

**Results and discussion:**

In 21 TAAD patients we found 22 rare variants, 6 (27.3 %) of these were previously reported, and 16 (73.7 %) were novel. Based on segregation data, functional analysis and software estimations we assumed that three of novel variants were causative, nine likely causative. Remaining four were classified as of unknown significance (2) and likely benign (2). In all, 9 (17.6 %) of 51 probands had a positive result when considering variants classified as causative only and 18 (35.3 %) if likely causative were also included. Genotype-positive probands (n = 18) showed shorter mean event free survival (41 years, CI 35–46) than reference group, i.e. those (n = 29) without any plausible variant identified (51 years, CI 45–57, p = 0.0083). This effect was also found when the ‘genotype-positive’ group was restricted to probands with ‘likely causative’ variants (p = 0.0092) which further supports pathogenicity of these variants. The mean event free survival was particularly low (37 years, CI 27–47) among the probands with defects in the TGF beta signaling (p = 0.0033 vs. the reference group).

**Conclusions:**

This study broadens the spectrum of genetic background of thoracic aneurysms and dissections and supports its potential role as a prognostic factor in the patients with the disease.

**Electronic supplementary material:**

The online version of this article (doi:10.1186/s12967-016-0870-4) contains supplementary material, which is available to authorized users.

## Background

Thoracic aortic aneurysms (TAA) are a significant cause of morbidity and mortality. The actual incidence is hard to estimate as the disease usually remains silent until an acute aortic dissection (AAD) or rupture occurs. Incidence of AAD is assessed at 2–3.5/100,000 inhabitants/year in the US [1]. It is also evaluated that individual lifetime risk of rupture or dissection reaches 34 % by the time aorta achieves a diameter of 6 cm [2].

There are two major types of TAAs: sporadic (degenerative/atherosclerotic) and hereditary thoracic aortic aneurysms and dissections (TAAD). Familial form is found in 20–40 % of TAAD cases when at least two subjects within single family are diagnosed with TAAD [3, 4]. TAAD may be a part of well-characterized genetic syndromes such as Marfan syndrome (MFS), Loeys–Dietz syndrome (LDS), vascular type of Ehlers–Danlos syndrome (EDS), Shprintzen–Goldberg syndrome—all named syndromic TAAD. However most TAAD patients do not meet the criteria of those syndromes and are defined as as non-syndromic TAAD [5].

After the discovery of the defect in fibrillin 1 gene (*FBN1*) as the mechanism underlying pathogenesis of MFS, and the identification of first causative mutation in 1991 [6–8], there are currently over 1300 mutations in HGMD database referred to MFS and over 3000 entries in UMD-FBN1 mutations database (http://www.umd.be/FBN1/). In 2003 Kroes et al. [9] related EDS to mutations in *COL3A1*, the following year Mizuguchi et al. [10] associated *TGFBR2* mutations with marfanoid phenotype, and finally in 2005 Loeys and colleagues distinguished and characterized LDS as the separate entity caused by defects in the *TGFBR1* and *TGFBR2* genes [11]. Soon followed the discoveries of genes responsible for non-syndromic TAAD [12, 13]. Autosomal dominant transmission of the TAAD has been proven in vast majority of patients with the disease, although in some probands with healthy parents, de novo mutations occur.

Introduction of next-generation sequencing (NGS) enabled efficient and cost-effective examination of all coding genes (whole exome sequencing, WES) or chosen gene panels.

Aim of the study was to define the diagnostic yield of NGS in 51 TAAD patients, to search for novel mutations in TAAD-associated genes, as well as genotype-phenotype correlations within families of TAAD patients.

## Methods

### Patients and consent

The study cohort was chosen from all index patients referred with the diagnosis of TAAD for clinical genetic testing from 2012 to 2014 to the Unit for Screening Studies in Inherited Cardiovascular Diseases, Institute of Cardiology and comprised 51 unrelated patients and 64 relatives of genotype positive patients. Patients with family history of aortic disease, early onset of aortic disease, suspected connective tissue disorders were included primary. In all patients a three-to-four generation pedigree was drawn and the data on the presence of TAAD, and other diseases in the family were collected. Every effort was made to review medical data on deceased subjects to confirm familial form of TAAD. For patients suspected of MFS and their relatives revised Ghent criteria were used [14] and a detailed questionnaire was applied to define the involvement of other systems and organs. Systemic score for each patient was calculated with web calculator http://www.marfan.org/resources/professionals/marfandx. In addition, web questionnaires were used to assess systemic features of LDS http://www.loeysdietz.org/ and Sphrintzen–Goldberg syndrome [15]. With regard to cardiovascular system, all patients had Doppler echocardiographic study and CT scan of the entire aorta. In particular, we collected the following data: age at diagnosis of all features, namely of the presence of TAA, the history of AAD, the presence of aortic arch and descending aorta dissection, the prophylactic surgery for thoracic aorta surgery (aortic root, ascending aorta). Indications for planned surgery relied on available guidelines and evolved with time. Definition of aortic root dilatation and ascending aorta dilatation were based on echocardiography with calculation of Z-score for aortic root using Web calculator http://www.marfan.org/resources/professionals/marfandx. According to guidelines, patients were followed-up with serial examinations by two-dimensional echocardiography and/or CT scan of the aorta. Both acute aortic dissection and first planned aortic surgery were considered aortic events. Data concerning mitral valve included presence of mitral valve prolapse (MVP) and mitral regurgitation (MR) on echocardiography. Diagnosis for MVP was based upon published criteria [16]. Familial disease was defined as the presence of >1 patient with TAAD in the family. This study was approved by the Bioethics Committee in the Institute of Cardiology (ref. no. 1273). All participants of the study gave an informed written consent including specific consent to genetic testing and permission to publish the results.

### Genetic testing

DNA was extracted from the peripheral blood by standard salting out method and from buccal swab using Maxwell^®^ 16 instrument with DNA IQ™ Casework Pro Kit (Promega).

In all probands sequencing was performed on HiSeq 1500. In 29 patients whole exome sequencing (WES) was performed, and in 22 TruSight One (TSO) sequencing panel (Illumina, San Diego, CA, USA) was used. WES sequencing libraries were prepared using TruSeq Exome Enrichment Kit (Illumina, San Diego, CA, USA) or Nextera Rapid Capture Exome (Illumina, San Diego, CA, USA) as described previously  [17].

We considered variants located in the coding or splicing regions of one of established TAAD genes with autosomal dominant transmission pattern (Table 1), of frequency no greater than 0.001 in all of three databases (1000Genomes, ESP and ExAC). Mutation was considered novel when absent from HGMD database (release 2015.1). To assess pathogenicity of novel variants we used algorithms supplied by ANNOVAR software (http://annovar.openbioinformatics.org/en/latest/user-guide/filter/#-lrt-annotation). Thus identified variants were followed-up with Sanger sequencing using a 3130xL Genetic Analyzer (Applied Biosystems, Foster City, CA, USA) and BigDye Terminator v1.1 Cycle Sequencing Kit (Applied Biosystems) according to the manufacturer’s instructions. The results were analyzed with Mutation Surveyor 3.30 Software (SoftGenetics). List of primers specific to each variant is available in Additional file 1. Amplification and Sanger sequencing of the beginning of exon 1 of *SKI* because of its CG-rich content was based on modification of methods published by Naz and Fatima [18]. Details are available in Additional file 1.

**Table 1:** List of analyzed genes

Gene	*OMIM*#
*ACTA2*	102620
*COL3A1*	120180
*FBN1*	134797
*MYH11*	160745
*MYLK*	600922
*SKI*	164780
*SMAD3*	603109
*TGFB2*	190220
*TGFBR1*	190181
*TGFBR2*	190182

Once mutation was identified, screening was offered to consenting relatives. Whenever possible we looked for co-segregation in the TAAD families.

### Statistical analysis

All results for categorical variables were presented as numbers and percentages and for continuous variables as mean and standard deviation (SD). Kaplan–Meier survival curves were used to compare event-free survival between genotype positive and negative patients. All tests were two-sided with the significance level of p < 0.05. Calculations were performed with *SPSS* package.

## Results and discussion

### Clinical findings

Table 2 shows summarized clinical characteristics of the study group. Mean age at the time of genetic inquest in the study group was 44 ± 13.8 years and 35 (68.6 %) patients were male. More than two-thirds of TAAD patients (n = 36, 70.6 %) had aortic events. Namely in 17 (33.3 %) patients AAD was first symptom of the disease at mean age of 40.1 years, and 19 (37.3 %) had planned aortic surgery at mean age of 44 years as first procedure. The remaining 15 (29.4 %) patients were diagnosed with TAA at mean age 39.9 years, however they did not meet criteria for surgical correction. Six patients after first operation for AAD required another surgical procedure during follow-up.

**Table 2:** Clinical characteristics of the study group, n = 51

*Clinical characteristics of the study group*	
Age at the genetic inquest (mean ± SD, years)	44.8 ± 13.8
Male sex (n, %)	35 (68.6)
AAD (n, %)	17 (33.3)
Age at AAD (mean ± SD, years)	40.1 ± 13.0
Planned TAA surgery (n, %)	25 (49)
Age at planned TAA surgery (mean ± SD, years)	42.2 ± 17
Planned TAA surgery—first procedure in the patient (n, %)	19 (37.3)
Age at the planned TAA surgery- first procedure in the patient (mean ± SD, years)	44 ± 16.8
TAA with no criteria for surgery (n, %)	15 (29.4)
Age at last examination (mean ± SD, years)	39.9 ± 14.9
Suspected MFS (n, %)	13 (25.5)
Familial TAAD	25 (49)
*Associated structural abnormalities (n,* *%)*	
BAV	11 (21.6)
CoA + BAV	1 (1.9)
ASD and LVNC	1 (1.9)
*Other CV diseases (n,* *%)*	
Stroke	3 (5.8)
CAD	6 (11.7)
Peripheral artery aneurysms	3 (5.8)
Hypertension	33 (64.7)
*Other diseases (n,* *%)*	
Mild-to-moderate intellectual disability	2 (3.9)
Rheumatid arthritis	1 (1.9)

In 13 patients with TAAD genetic examination was made to confirm/exclude the diagnosis of MFS. In the whole group co-existent abnormalities included: CoA+ BAV− 1 patient, isolated BAV− 11 patients, LVNC + ASD in one patient. Peripheral artery aneurysms were present in 3 (5.8 %) patients. A history of stroke was present in 3 (5.8 %), CAD in 6 (11.7 %) patients. More than half of the all study group 33 (64.7 %) patients were hypertensive. Familial disease was found in 25 (49 %) of TAAD patients. In addition, mild to moderate intellectual disability was present in 2 patients (3.9 %), rheumatoid arthritis in 1 (1.9 %).

### Mutation analysis and diagnostic sensitivity

**Fig. 1:**
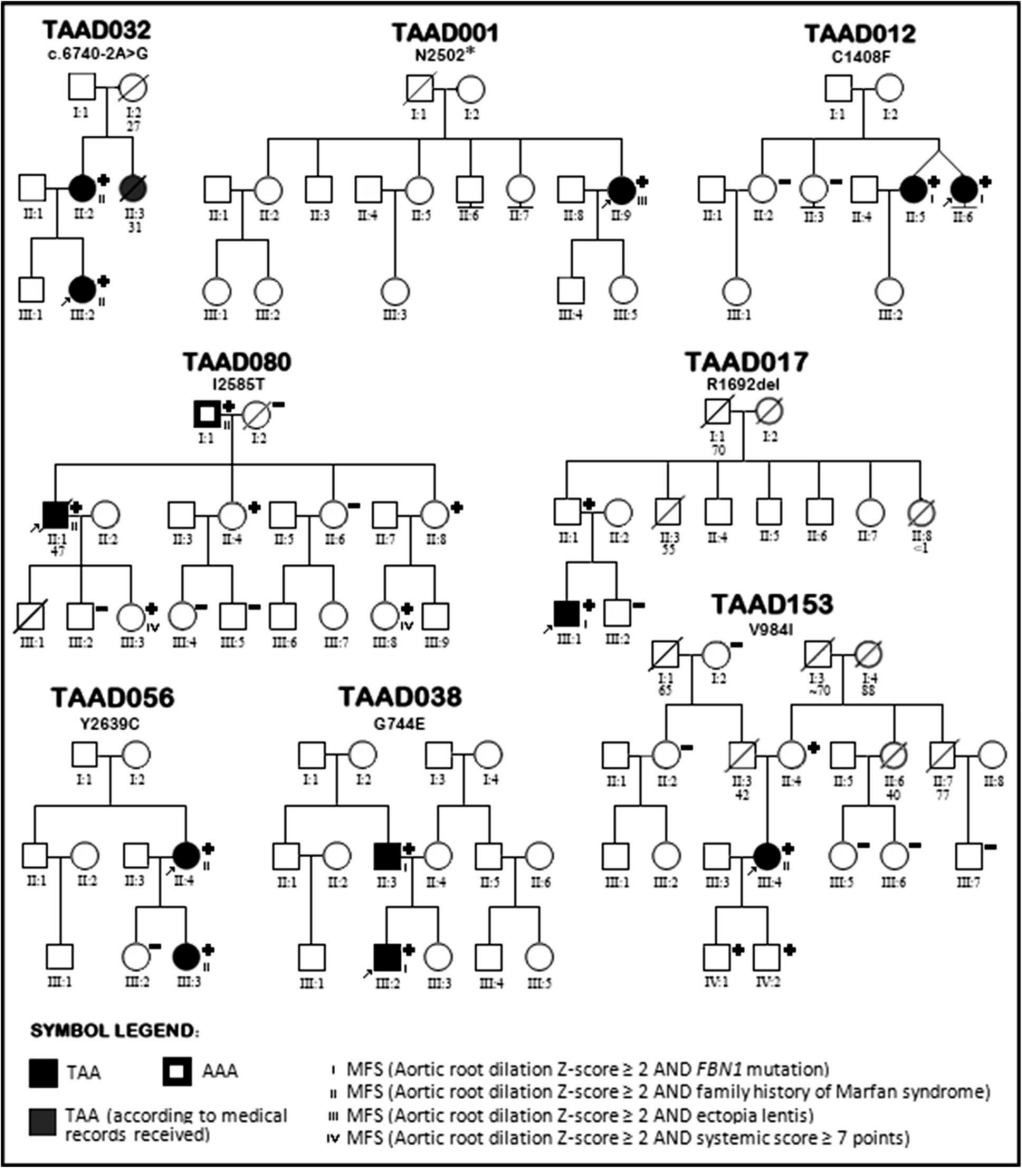
Pedigrees of families with fibrillinopathies

**Fig. 2:**
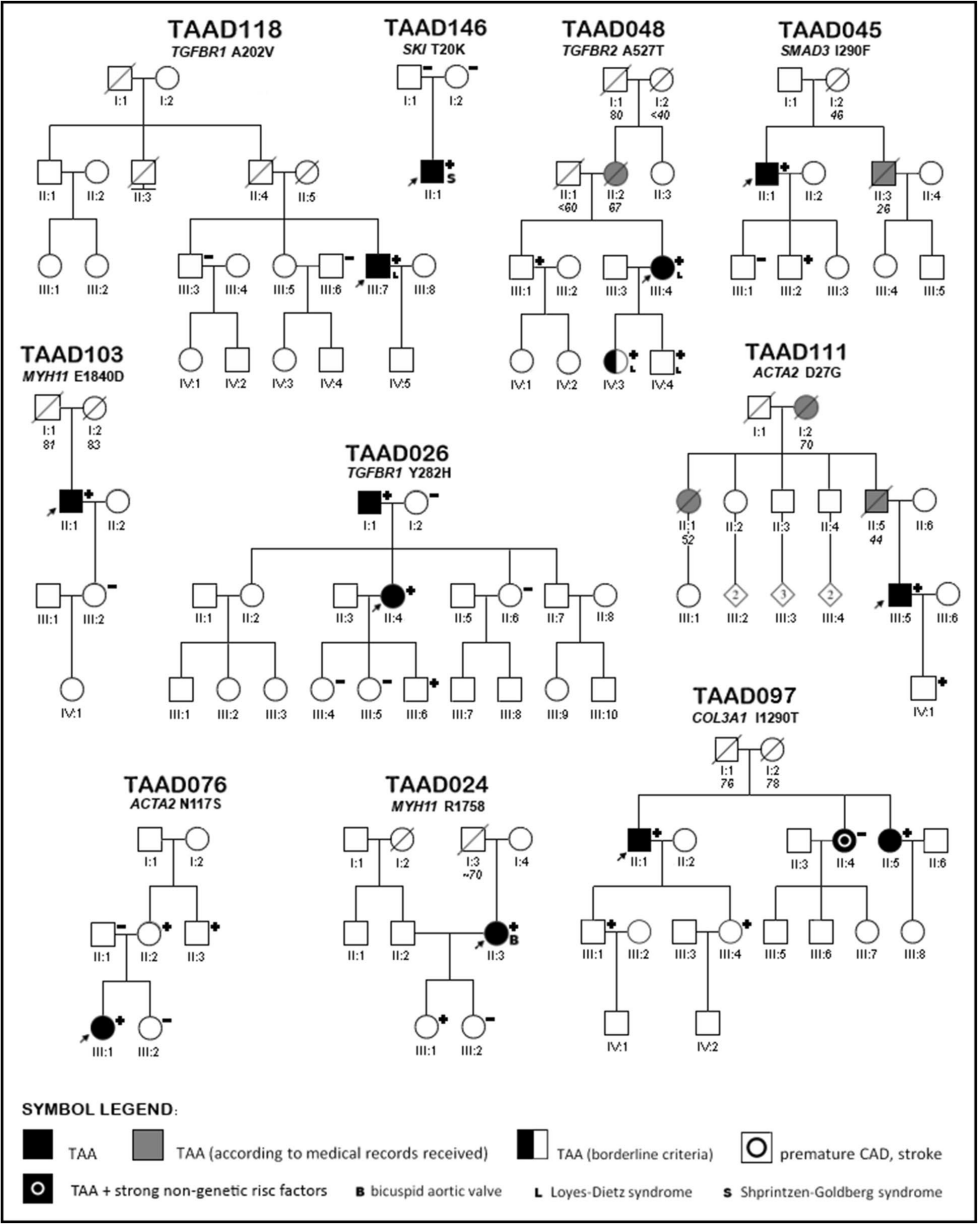
Pedigrees of families with mutations in *ACTA2, COL3A1, TGFBR1, MYH11, SKI, SMAD3, TGFBR1* and *TGFBR2* genes

In a total of 51 analyzed individuals we found 22 rare variants in a given panel of genes (Table 3). Six (27.3 %) of these were previously reported as pathogenic in TAAD patients. Among novel variants, one was a de novo event, and two were gene-disrupting variants. Together those nine variants were classified as causative. Another nine novel missense variants were considered likely causative as segregation analysis (Figs. 1, 2) either supported their causative effect and/or pathogenicity estimations strongly suggested their damaging effect. Also, in our study we classified as likely pathogenic a variant which was previously reported as pathogenic but was detected only in *cis* configuration with another (splice site) variant. Two missense sequence changes were considered as variants of unknown significance (VUS) since there was insufficient data for segregation study and software estimations gave ambiguous results. Two variants were classified as likely benign since they showed lack of co-segregation with the disease. Overall, 9 (17.6 %) of 51 probands had a positive result when considering variants classified as causative only and 18 (35.3 %) if likely causative variants were also included. Detailed clinical characteristics of genotype positive probands and family members are shown in Tables 4 and 5.

**Table 3:** Rare variants identified in our cohort and associated nomenclature, classification and database frequency

No	Family	Gene	Nucleotide	Protein	Transcript	Domain	Classification	References	ExAC	ESP
1	TAAD032	*FBN1*	c.6740-2A>G	Splice site	NM_000138.4	cbEGF-like	Causative	This study	0	0
2	TAAD001	*FBN1*	c.7502_7503insA	N2502*	NM_000138.4	cbEGF-like	Causative	This study	0	0
3	TAAD012	*FBN1*	c.4223G>T	C1408F	NM_000138.4	cbEGF-like	Causative	Tiecke et al. [22]	0	0
4	TAAD080	*FBN1*	c.7754T>C	I2585T	NM_000138.4	cbEGF-like	Causative	Liu et al. [48]	0	0
5	TAAD017	*FBN1*	c.5074_5076delAGA	R1692del	NM_000138.4	–	Causative	Comeglio et al. [29]	0	0
6	TAAD056	*FBN1*	c.7916A>G	Y2639C	NM_000138.4	cbEGF-like	Causative	Mátyás et al. [34]	0	0
7	TAAD038	*FBN1*	c.2231G>A	G744E	NM_000138.4	cbEGF-like	Likely causative	This study	0	0
8	TAAD153	*FBN1*	c.2950G>A	V984I	NM_000138.4	TB5	Causative	Grau et al. [38]	0.00005	0
9	TAAD118	*TGFBR1*	c.605C>T	A202 V	NM_004612.2	GS	Likely causative	This study	0	0
10	TAAD026	*TGFBR1*	c.844T>C	Y282H	NM_004612.2	Protein kinase	Likely causative	This study	0.00002	0
11	TAAD048	*TGFBR2*	c.1579G>A	A527T	NM_003242.5	Protein kinase	Causative	Frischmeyer-Guerrerio et al. [40]	0	0
12	TAAD146	*SKI*	c.59C>A	T20 K	NM_003036.3	R-SMAD binding	Causative	This study	0	0
13	TAAD045	*SMAD3*	c.868A>T	I290F	NM_005902.3	MH2	Likely causative	This study	0	0
14	TAAD111	*ACTA2*	c.80A>G	D27G	NM_001613.2	Actin family	Likely causative	This study	0	0
15	TAAD076	*ACTA2*	c.350A>G	N117S	NM_001613.2	Actin family	Likely causative	This study	0	0
16	TAAD024	*MYH11*	c.5273G>A	R1758Q	NP_002465.1	Coiled coil	Llikely causative	Zhu et al. [12]	0.0002	0.00023
17	TAAD103	*MYH11*	c.5499G>C	E1833D	NM_002474.2	Coiled coil	Likely causative	This study	0.0003	0.00054
18	TAAD157	*COL3A1*	c.2108C>T	P703L	NM_000090.3	Triple-helical region	VUS	This study	0	0
19	TAAD097	*COL3A1*	c.3869T>C	I1290T	NM_000090.3	NC1	Likely causative	This study	0	0
20	TAAD130	*MYLK*	c.608C>T	P203L	NM_053025.3	Ig-like C2-type 2	VUS	This study	0	0
21	TAAD027	*MYLK*	c.1133G>A	R378H	NM_053025.3	–	Likely benign	This study	0.0001382	0.00008
22	TAAD048	*MYLK*	c.2069C>T	T690 M	NM_053025.3	Ig-like C2-type 5	Likely benign	This study	0.000008	0.00008

**Table 4:** Clinical and genetic characteristics of syndromic TAAD patients and related mutation carriers

Family	Position on pedigree-status	Sex	Age	Ectopia lentis	Systemic score	CVS-involvement of the aorta	Aortic root	Z-score	IA	Extension	Age at surgery^a^	Type of surgery^a^	CVS-other	Type of syndrome	Gene		Mutation
TAAD032	III:2-proband	F	21	0	4	TAA	36	2.6	0	R	NA			MFS	*FBN1*	c.6740-2A > G	
TAAD032	II:2-mother	F	53	0	3	TAA	39	2.7	1	R	NA			MFS	*FBN1*	c.6740-2A > G	
TAAD001	II:9-proband	F	43	0	7	AAD	47	5.7	1	R, As, Ar, D, Ab	39	SC + A		MFS	*FBN1*	p.N2502X	
TAAD012	II:6-proband	F	32	1	2	TAA	50	7.6	0	R	32	Dav		MFS	*FBN1*	p.C1408F	
TAAD012	II:5-twin sibling	F	32	1	5	TAA	51	8.1	0	R	28	Dav		MFS	*FBN1*	p.C1408F	
TAAD080	II:1-proband	M	48	0	NE	AAD	Surgery		0	R, As, Ar, D	42	AVR + SC		MFS	*FBN1*	p.I2585T	
TAAD080	I:1-father	M	76	0	0	AAA	36	0.0	1	No	60	ABBG		MFS	*FBN1*	p.I2585T	
TAAD080	II:4-sister	F	48	0	0	N	33	0.8	0	No	NA			MFS mutation carrier	*FBN1*	p.I2585T	
TAAD080	II:8-sister	F	37	0	3	N	34	1.7	0	No	NA			MFS mutation carrier	*FBN1*	p.I2585T	
TAAD080	III:8-niece	F	16	0	2	N	30	0.8	1	No	NA			Potential MFS	*FBN1*	p.I2585T	
TAAD080	III:3-daughter	F	15	0	2	N	33	2.1	0	R	NA			Potential MFS	*FBN1*	p.I2585T	
TAAD017	III:1-proband	M	42	1	6	TAA	48	4.2	1	R, As	NA			MFS	*FBN1*	p.R1692del	
TAAD017	II:1-father	M	66	0	4	N	42	1.4	1	R	NA			MFS mutation carrier	*FBN1*	p.R1692del	
TAAD056	II:4-proband	F	61	0	2	TAA	57	9.2	3	R, As, Ar	58	B		MFS	*FBN1*	p.Y2639C	
TAAD056	III:3-daughter	F	28	0	5	TAA	44	4.8	0	R	NA			MFS	*FBN1*	p.Y2639C	
TAAD038	III:2-proband	M	22	0	5	TAA	41	3.2	0	R	NA		MVP, MR 2+	MFS	*FBN1*	p.G744E	
TAAD038	II:3-father	M	48	0	3	TAA	43	2.4	0	R	NA		MVP, MR 1+	MFS	*FBN1*	p.G744E	
TAAD153	II:2-proband	F	48	0	1	TAA	46	5.7	3	R, As, Ar	48	B		MFS	*FBN1*	p.V984I	
TAAD153	I:3-mother	F	72	0	5	N	35	1.2	1	No	NA			MFS mutation carrier	*FBN1*	p.V984I	
TAAD153	IV:1-son	M	22	0	0	N	32	−0.4	0	No	NA			MFS mutation carrier	*FBN1*	p.V984I	
TAAD153	IV:2-son	M	21	0	0	N	29	−1.2	0	No	NA			MFS mutation carrier	*FBN1*	p.V984I	
TAAD108	proband	M	18	0	10	TAA	Surgery		1	R	19	B		MFS	Not found	NA	
TAAD118	III:7-proband	M	26	0	8	TAA	Surgery		0	R, As	18	D		LDS	*TGFBR1*	p.A202 V	
TAAD048	III:4-proband	F	42	0	0	AAD	Surgery		1	R, As, Ar, D, Ab	40	SC		LDS	*TGFBR2*	p.A527T	
TAAD048	III:1 - brother	M	43	0	4	N	36	0.7	0	no	NA			LDS mutation carrier	*TGFBR2*	p.A527T	
TAAD048	IV:3-daughter	F	19	0	7	Borderline TAA	36	2.8	0	R	NA			LDS	*TGFBR2*	p.A527T	
TAAD048	IV:4-son	M	16	0	7	Borderline TAA	38	2.7	0	R	NA			LDS	*TGFBR2*	p.A527T	
TAAD146	II:1-proband	M	26	0	10	TAA	49	5.6	1	R	NA		MVP, MR 2+	SGS	*SKI*	p.T20 K	

*AAA* abdominal aortic aneurysm, *AAD* acute aortic dissection, *Ab* abdominal aorta, *ABBG* aorto–bifemoral bypass grafting, *Ar* aortic arch, *AVR* aortic valve replacement, *B* Bentall procedure, *CVS* cardiovascular system, *Dav* David procedure, *D* thoracic descending aorta, *F* female, *LDS* Loyes-Dietz syndrome, *M* male, *MR* mitral regurgitation (*0* none, *1* mild, *2* moderate, *3* severe), *MFS* Marfan syndrome, *N* normal echocardiographic study, *NA* not applicable, *NE* not examined, *R* aortic root, *SC* supracoronary ascending aortic replacement, *SC* *+* *A* supracoronary and arch prosthesis, *SGS* Shprintzen–Goldberg syndrome, *TAA* thoracic aortic aneurysm

^a^Relates to primary surgical intervention

**Table 5:** Clinical and genetic characteristics of nonsyndromic mutation carriers

Family	Position on pedigree-status	Sex	Age	CVS involvement of the aorta	Aortic Root	Z-score	Extension	Age at surgery	Type of surgery^a^	Cardiovascular system-other	Gene	Protein
TAAD026	II:4-proband	F	52	AAD	Surgery		R, As, Ar, D, Ab	46	SC		*TGFBR1*	p.Y282H
TAAD026	I:1-father	M	77	TAA	47	3.8	R, As, Ar	NA	NA	CAD	*TGFBR1*	p.Y282H
TAAD026	III:6-son	M	15	N	30	0.5		NA	NA		*TGFBR1*	p.Y282H
TAAD045	II:1-proband	M	47	AAD	Surgery		R, As, Ar, D, Ab	35	SC		*SMAD3*	p.I290F
TAAD045	III:2-son	M	21	N	37	1.6		NA	NA		*SMAD3*	p.I290F
TAAD111	III:5-proband	M	41	AAD	Surgery		As, Ar, D, Ab	29	SC		*ACTA2*	p.D27G
TAAD111	IV:1-son	M	19	N	31	−0.1		NA	NA		*ACTA2*	p.D27G
TAAD076	III:1-proband	F	29	AAD	36	2.7	R, Ad, Ab	20	SGI		*ACTA2*	p.N117S
TAAD076	II:2-mother	F	55	N	30	0.9		NA	NA		*ACTA2*	p.N117S
TAAD076	II:3-mat uncle	M	57	N	36	NE		NA	NA	Stroke at 39 y, MI at 42y	*ACTA2*	p.N117S
TAAD024	II:3-proband	F	49	TAA	Surgery		As, Ar	43	SC	BAV	*MYH11*	p.R1758Q
TAAD024	III:1-daughter	F	24	N	30	0.6		NA	NA		*MYH11*	p.R1758Q
TAAD103	II:1-proband	M	62	TAA	Surgery		R, As, Ar onset	59	Y + AVR	BAV	*MYH11*	p.E1840D
TAAD097	II:1-proband	M	55	AAD	Surgery		As, Ar, D, Ab	40	SC + A		*COL3A1*	p.I1290T
TAAD097	II:5-sister	F	50	TAA	44	3.4	R, As				*COL3A1*	p.I1290T
TAAD097	III:1-son	M	33	Borderline TAA	42	2.0	R				*COL3A1*	p.I1290T
TAAD097	III:4-daughter	F	28	N	32	1.1					*COL3A1*	p.I1290T

*AAD* acute aortic dissection, *Ab* abdominal aorta, *Ar* aortic arch, *As* ascending aorta, *AVR* aortic valve replacement, *BAV* bicuspid aortic valve, *CAD* coronary artery disease, *CVS* cardiovascular system, *D* thoracic descending aorta, *F* female, *HT* arterial hypertension, *M* male, *MI* myocardial infarction, *N* normal echocardiographic study, *NA* not applicable, *NE* not examined, *R* aortic root, *SC* supracoronary ascending aortic replacement, *SC* *+* *A* supracoronary and arch prosthesis, *SGI* stent graft implantation, *TAA* thoracic aortic aneurysm, *Y* Yacoub procedure

^a^Relates to primary surgical intervention

There were 3 recently published studies which applied NGS for molecular diagnosis of both syndromic and non-syndromic TAAD. Wooderchak-Donahue et al. used a panel of 10 genes in the group of 175 patients. Overall they found 51 rare variants, 10 % of patients had variants classified as pathogenic and 18 % had VUS (CNV’s found by SGH-array not included) [19]. Proost et al. used panel of 14 genes to study 55 individuals and reported 15 causative variants and 6 VUS achieving 27 % clinical sensitivity [20]. Ziganshin et al. [21] used WES to study 102 patients, of whom 72.5 % had no potentially relevant alterations in 21 genes analyzed. Only 4 % of participants had variants which authors considered as deleterious and 22 % had previously unreported VUS. In comparison with the above mentioned studies the diagnostic yield in our study (i.e. 35.3 %) was relatively high which may be possibly caused by a relatively severe disease in our cohort (see below the section on genotype phenotype correlations and the data indicating that causative mutations are more often detected in severe disease). The generally low diagnostic yield (≪50 %) observed in all the studies indicates that in the major proportion of patients the disease is caused by factors (genetic or environmental) which are at present unknown.

### Genotype phenotype correlations

**Fig. 3:**
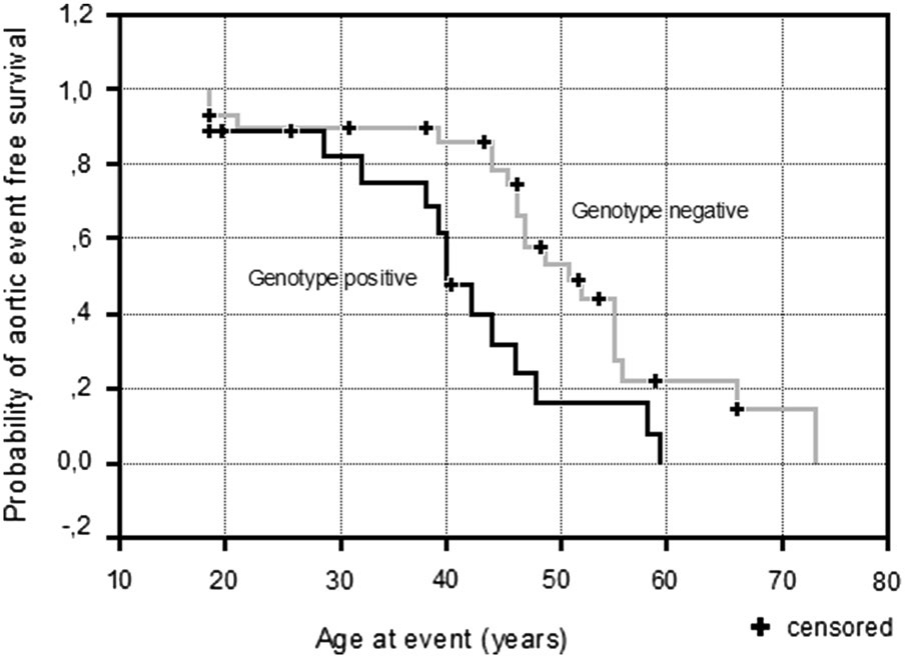
Kaplan–Meier analysis of event free survival in TAAD in probands with variants classified as causative/likely causative vs. those without any candidate variants identified (Log-Rank Chi- square 6.97, p = 0.0083)

Kaplan–Meier survival curve was constructed to compare the event-free survival between probands defined as ‘genotype-positive’ (i.e. those with causative/likely causative variants) and ‘genotype-negative’ reference group (i.e. those without any plausible variant identified, including VUS and likely benign variants). Aortic events were defined as acute aortic dissection or first planned aortic surgery. Genotype-positive probands showed shorter mean event free survival (41 years, CI 35–46) than those who were genotype-negative (51 years, CI 45–57, p = 0.0083, Fig. 3). Interestingly, this effect was also found when the ‘genotype-positive’ group was restricted to probands with variants classified as ‘likely causative’: 37 years (CI 28–47) vs. 51 years (CI 45–57, p = 0092, Additional file 1: Figure S1). This latter observation argues that majority (if not all) of the variants classified as ‘likely pathogenic’ are functionally relevant. The generally faster disease progression among subjects with causative/likely causative variants suggests that the currently known TAAD loci are those associated with the most severe disease.

When we considered functional pathway altered by mutation, we found that the mean event free survival was 37 years (CI 27–47, p = 0.0033 vs. the ‘genotype-negative’ reference group, Additional file 1: Figure S2) among the probands with defects in the TGF beta signaling (i.e. those with *TGFBR1*, *TGFBR2*, *SMAD3* or *SKI* mutations). The mean event free survival for probands with *FBN1* mutations was 45 years (CI 36–53, NS vs. the reference), whereas for the remaining probands with causative/likely causative variants (i.e. those with mutations in *ACTA2, MYH11* or *COL3A1*) it was 38 years (CI 24–52, NS vs. the reference). The particularly fast disease progression associated with defects in TGF beta signaling has been observed previously [5].

Pedigrees of families with mutations in *FBN1* are shown in Fig. 1 and those with the remaining mutations in Fig. 2. Detailed clinical characteristics of genotype positive probands and their family members are presented in Tables 4 and 5.

### FBN1

Similar to previous reports [19, 20] *FBN1* variants were the most abundant in our study group. Among eight *FBN1* mutations identified, 3 were novel: c.6740-2A>G disrupting splice site acceptor, c.7502_7503insA (p.N2502*) introducing stop codon at exon 61, and p.G744E affecting calcium binding epidermal growth factor-like domain (cb EGF-like).

The proband carrying *FBN1* c.6740-2A>G (family TAAD032) is a 21-year-old woman who was suspected of MFS because of TAA with Z-score of 2.6 and mild systemic features with systemic score of four points. The proband’s mother was 53-year-old woman, with positive family history of AAD (her sister died at age of 31 in 6th month of pregnancy, and her mother died suddenly at age of 27 years). She had TAA with Z-score 2.7, systemic score of 3 points, no ocular involvement. In summary, without genetic examination the diagnosis of MFS would not be possible in the family.

p.N2502* was found in a 43-year-old patient (family TAAD001), suffering from AAD (Stanford, type A) at age of 39 years, without diagnosis of MFS, without remarkable family history. Based on clinical features, she met systemic criteria (seven points) for the diagnosis of MFS. Family history was negative. MFS could be diagnosed on the basis of TAAD and systemic score, identification of *FBN1* truncating variant confirmed the clinical diagnosis.

*FBN1* p.G744E variant was identified in 22-year-old proband and his 48-year-old father with suspected MFS (family TAAD038). None of them had ectopia lentis or met systemic criteria for the diagnosis of MFS. The proband had surgical correction of pectus excavatum at age of 14 years, and had significant myopia (−9D). His major problem was prolapse of both leaflets of the mitral valve with significant mitral insufficiency, enlarged left ventricle (LVEDD 62 mm) with preserved left ventricular systolic function (LVEF 64 %). In addition dilated aortic root (Z-score 3.2) was present at age of 22 years. His father with myopia of −3D also had mitral valve prolapse, however with mild mitral insufficiency and dilated aortic root with Z-score of 2.4. The disease is more aggressive in the proband than in his father.

Four of previously reported *FBN1* mutations (p.C1408F, p.I2585T, p.R1692del, and p.Y2639C) were located in cb EGF-like domains, and one (p.V984I) in TGFβ Binding-Protein-Like Domain 5 (TB5).

p.C1408F has been once reported previously [22], however, there are two more reports of another alteration in the same cysteine residue in MFS patients [23, 24] indicating that the position may be a mutational hot spot. p.C1408F was found in monozygotic affected female twins from our study group (family TAAD012). Both twins had ectopia lentis, and required prophylactic aortic surgery due to TAA at age of 28 and 32 years. The variant is probably de novo in the family as two sisters were free of mutation and parents were without any remarkable history.

*FBN1* p.I2585T first reported by Liu et al. is one of the most recurrent MFS-causing mutations. According to the UMD-FBN1 mutations database this variant was detected in 30 MFS patients of either Asian or Caucasian descent [24–31]. We found a remarkable clinical variability in the family with six mutation carriers in three generations (family TAAD080). Involvement of the cardiovascular system was present in the proband (AAD of the thoracic aorta at age of 42 years, rapidly progressive after emergency surgery) and in the proband’s father (no TAA, however aneurysm of the abdominal aorta was present corrected surgically at age of 60 years). Two proband’s sisters had only skeletal involvement with scoliosis, in two mutation carriers of the youngest generation we diagnosed potential MFS.

There are two reports of MFS patients with *FBN1* p.R1692del mutation. The first concerns a patient after vascular and/or valve surgery [32] and the second a 7 years old child with ectopia lentis and minor skeletal and no cardiovascular involvement [29]. Larger in frame deletion in the same exon (5074_5097del) was reported in Weill-Merchesani syndrome [33]. In our family with two mutation carriers (family TAAD017), only proband 42-year-old met criteria for classic MFS with both ectopia lentis and TAA. His father did not have either ocular involvement or TAA but as he is a mutation carrier, he can be diagnosed as MFS genetically predisposed.

*FBN1* p.Y2639C was first reported by Matyas et al. [34], since then it has been found in several other studies on MFS and related disorders [25, 35, 36] including a report describing 19 individuals carrying p.Y2639C in a single family who showed extensive clinical variability from minor skeletal abnormalities to full diagnostic criteria for MFS with TAAD and mitral valve prolapse [37]. In our family (TAAD056) the proband was 61 years old female diagnosed at age of 58 years because of a history of exertional dyspnea, severe aortic insufficiency with aortic root dilatation of 57 mm and generalized hypokinesis of the left ventricle with LVEF of 46 %. She underwent Bentall procedure which led to restoration of left ventricular function. Her daughter had TAA diagnosed at the age of 28 years with Z-score of 4.8. None of them had ectopia lentis or reached systemic score of seven points, therefore MFS could only be diagnosed based on genetic examination and the presence of TAA.

*FBN1* p.V984I is located in so-called neonatal region of *FBN1* where all reported mutations associated with neonatal MFS and the majority of point mutations associated with atypically severe presentations were found [22] although it has been previously found also in a male patient with classical MFS [38]. Our 48-year-old female proband (TAAD153) has a history of Bentall procedure due to severe aortic insufficiency and dilatation of the aortic root and ascending aorta. Interestingly, the proband had also tortuosity of the vessels and impaired healing of wounds. The diagnosis of MFS in the proband was possible only after genetic examination. Among the remaining three mutation carriers (proband’s mother and proband’s sons) none had TAA. Proband’s mother had minor skeletal involvement.

### TGFBR1 and TGFBR2

We found three rare variants in genes coding TGFβ receptors, all located within serine threonine kinase domain of both proteins where the most of pathogenic missense variants are being found [39]. p.A527T in *TGFBR2* has been reported previously in LDS patient [40]. Two novel variants in *TGFBR1* (p.A202V and p.Y282H) we assumed to be likely causative.

*TGFBR1* p.A202V was identified in 26 years old male with MFS diagnosed on the basis of systemic criteria and the presence of TAA (family TAAD118). His father died of AAD at age of 40 years. There was no ocular involvement. The patient underwent David’s procedure at age of 18 years. In addition dilation of the proximal parts of both coronary arteries, the brachiocephalic trunk, and the pulmonary artery were found. At age of 6 years he was operated on because of horseshoe kidney.

*TGFBR1* p.Y282H was identified in 52 years old female hypertensive patient (family TAAD026), who suffered from AAD type A at the age of 46 years, with chronic dissection of thoraco-abdominal aorta, without systemic features characteristic of MFS, with hyperopia (+7D). The variant came to her from her 77 years old father with TAA (aortic root 47 mm, Z-score 3.8), with coronary heart disease (post inferior MI), treated with CABG at the age of 67 years, and PCI at the age of 69 years. Her son is asymptomatic mutation carrier.

The proband carrying p.A527T in the *TGFBR2* gene is a 42-year-old woman (family TAAD048) with a history of type A AAD at the age of 40 and rapid progression following emergency operation (the implantation of an ascending aortic prosthesis in supracoronary position) requiring additional two-step procedures: (Bentall operation with the aortic arch replacement and descending thoracic aorta replacement 9 months later). The patient presents few features of LDS type 1 such as hypertelorism and cervical spine instability, no bifid uvula and no typical signs of MFS. At the age of 17 she underwent surgery for branchial cleft cyst, later she required repetitive surgery for recurrent umbilical hernia, lower limb varicose veins and hemorrhoids. She also suffers from allergic disease which is consistent with recently published study pointing to the strong predisposition to allergies among LDS patients due to altered TGFβ signaling [40]. MRI of the whole arterial tree revealed arterial tortuosity of the intracranial arteries. On repetitive CT thoraco-abdominal scans an aneurysm of the hepatic artery was also identified. The proband’s mother died at the age of 67 subsequently to type B aortic dissection. Both proband’s children tested positive for *TGFBR2* gene mutation. In the second decade of life they met systemic criteria for the diagnosis of MFS and had aortic root dilatation with Z-score 2.7 and 2.8, respectively. The proband’s brother, another mutation carrier, at age of 43 years had no aortic root dilation and mild involvement of other tissues features. At age of 41 he experienced spontaneous pneumothorax.

### SKI

De novo *SKI* p.T20K mutation has been found in a proband with clinical suspicion of Shprintzen–Goldberg syndrome (family TAAD146). So far, all discovered variants causing this syndrome occurred in exon 1, between amino acid residues 21 and 117. Mutations in this area (some of them recurrent) cluster between two regions: one encoding R-SMAD binding domain and the other encoding start of Dachshund-homology domain (DHD) [41]. This is the first report of SGS-causing variant affecting residue 20 which also is a part of R-SMAD binding domain emphasizing the role of this region of the gene in disease pathogenesis.

**Fig. 4:**
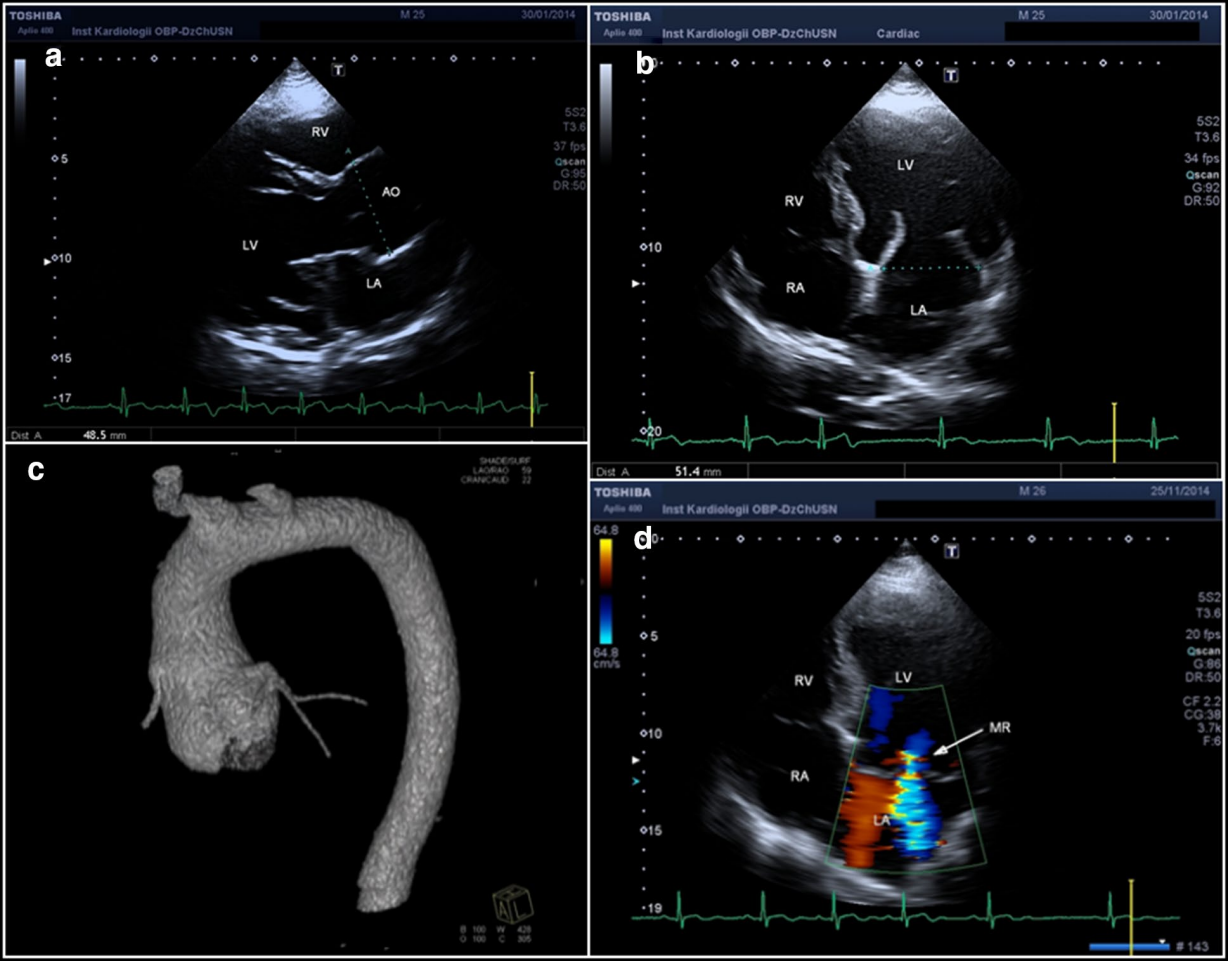
Cardiovascular imaging study in the patient with *SKI* p.T20 K variant. **a** 2D transthoracic echocardiography of parasternal long axis demonstrated aortic root aneurysm with Valsalva sinuses diameter of 48.5 mm.; **b** and **d** 2D transthoracic echocardiography in apical four chamber view in diastole. Enlarged left ventricle and left atrium with increased mitral annular diameter of 51.4 mm; **c** contrast-enhanced, ECG-gated CT, volume rendering (VR) image, the aneurysm of the ascending aorta; **d** *color flow* Doppler study revealed significant mitral regurgitation due to thickened and floppy mitral valve leaflets and widened mitral annulus

The 26 years old proband had TAA (aortic root size diameter 48.5 mm, Z-score 5.6), dilated pulmonary artery (31 mm), mesotelesystolic prolapse of both mitral leaflets with moderate mitral insufficiency, mitral ring of 51.4 mm, dilated left ventricle of 66 mm (LVEDD), dilated left atrium of 43 cm^2^ and LVEF of 55 % (Fig. 4). Systemic score for MFS was nine. Other abnormalities included the following: craniofacial findings (dolichocephaly, downslanting palpebral features, malar flattening, high narrow palate with prominent palatine ridges and retrognathia) and skeletal findings (dolichostenomelia, arachnodactyly, pectus carinatum, scoliosis, pes planus, foot and knee malposition). Of importance, joint hypermobility made it difficult for the patient to keep balance on the moving bus. Of the neurological anomalies moderate intellectual disability was found with the patient not able to perform any work. Other abnormalities included myopia (−4/−4.5D) along with astigmatism. In addition, the patient suffered from recurrent hypertrophy of the third tonsil that restricted his breathing, operated on four times.

### SMAD3

*SMAD3* p.I290F, identified in a male proband (family TAAD045) with AAD at the age of 47 years is a novel variant located in Mad homology 2 (MH2) domain highly conserved among different species and other SMAD proteins. Neighboring variants from this locus have been repeatedly reported in aneurysm-osteoarthritis syndrome [42, 43]. The proband who had a history of hypertension and suffered from AAD type A at the age of 35 years, died of complications of dissected abdominal aorta at the age of 47 years. The family history was positive for heart disease (mother died at the age of 46 years, and brother died at the age of 26 years because of aortic aneurysm). One of his sons, the mutation carrier, at the age of 21 years presented with hypertension and mildly dilated aortic root with Z-score 1.6. None of the mutation carriers had any history of osteoarthritis. However, elder of his sons, aged 23 years, free of the mutation and dilation of the aorta despite hypertension was operated on because of extraarticular, tenosynovial chondromatosis at age of 9 years. Furthermore his daughter, genetically not tested, at age of 18 years with normal aortic dimensions, also had tenosynovial chondromatosis.

### MYH11

The proband carrying *MYH11* p.E1840D (family TAAD103) was 62-year-old patient with BAV, TAA and a history of hypertension well controlled on two drugs, after planned procedure of aortic mechanical valve implantation SJM 25 mm plus supracoronary graft at age of 59 years, with a history of rheumatoid arthritis. No PDA on angio CT scan of the aorta. The patient’s daughter, aged 32 years was free of aortic disease and the mutation-free.

The 49-year-old proband carrying *MYH11* p.R1758Q (family TAAD1758) was diagnosed with BAV and TAA. At the age of 44 she underwent the supracoronary aortic graft implantation. At the age of 49 she was found to have nonprogressive aortic arch dilatation (42 mm). One of her two daughters is the asymptomatic carrier of the mutation.

**Fig. 5:**
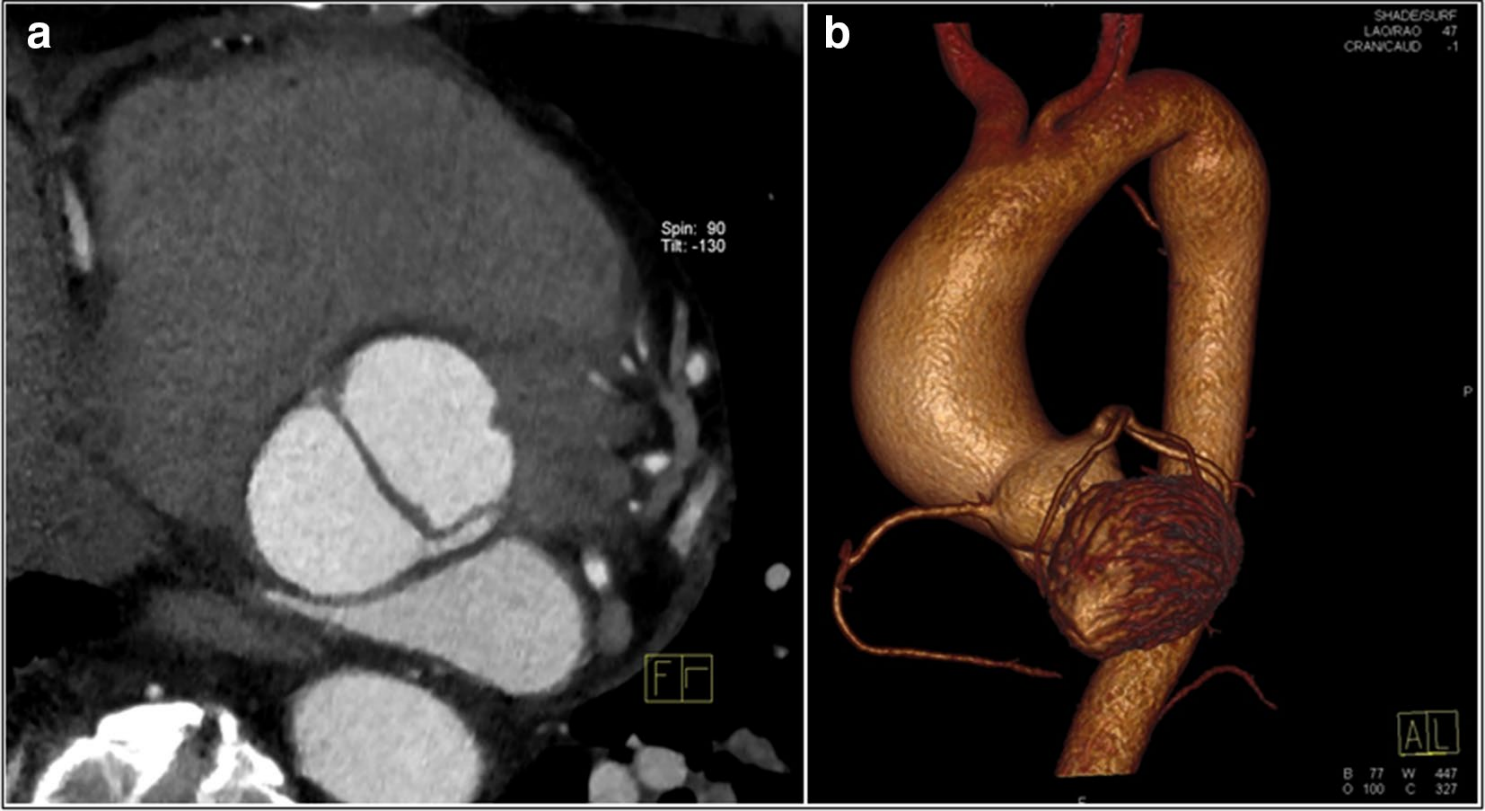
Cardiovascular imaging study in the patient with *MYH11* p.E1840D variant. **a** contrast-enhanced, ECG-gated CT, multiplanar reformatted image with reconstruction parallel to aortic valve shows bicuspid aortic valve in diastole; **b** volume rendering (VR) image, the aneurysm of the thoracic aorta: root of 43 mm, ascending aorta- of 52 mm (maximum dimension) and aorta before the origin of brachiocephalic trunk of 40 mm, farther arch dimension is normal of 28 mm

**Fig. 6:**
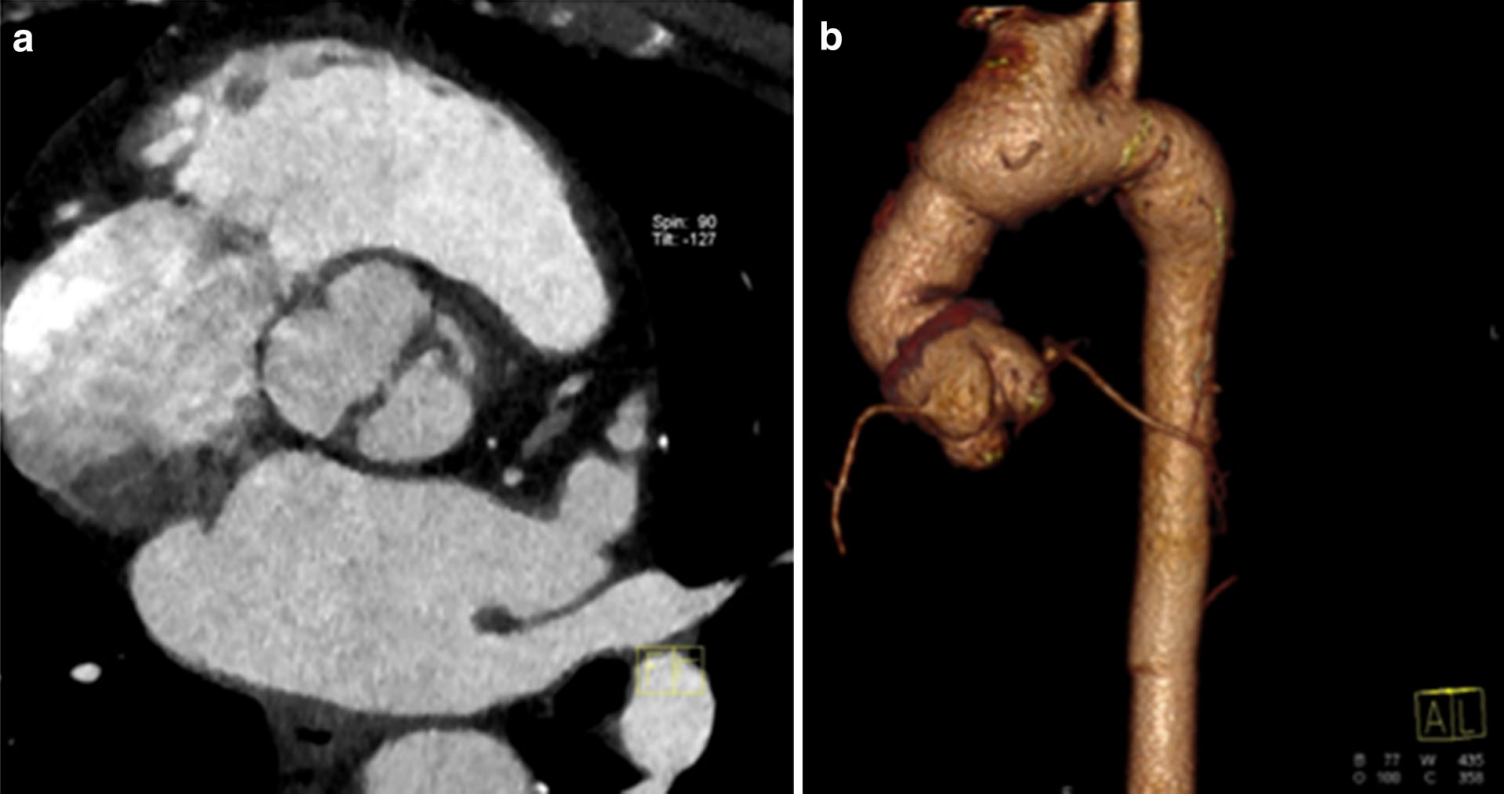
Cardiovascular imaging study in the patient with *MYH11* p.R1758Q variant. **a** contrast-enhanced, ECG-gate CT, multiplanar reformatted images with reconstruction parallel to aortic valve show bicuspid aortic valve in diastole; **b** volume rendering (VR) image shows supracoronary prosthesis and dilated aortic arch. Variant anatomy: common origin of brachiocephalic and left common carotid artery. Aortic arch diameter before the anomaly of 42 mm

Two *MYH11* mutations, identified in our probands: p.E1840D—a novel mutation and a previously reported p.R1758Q [12] are both localized in myosine tail. However, according to the previous report the p.R1758Q was identified only in the presence of another variant which resulted in skipping of exon 32 [12]. The deletion segregated with disease in studied family and was predicted to affect myosine tail domain’s coiled-coil structure and the assembly of myosin thick filaments. It was not clear whether the additional presence of p.R1758Q in some affected individuals had any effect [12]. Although *MYH11* mutations are described to cause TAA and PDA, families with incomplete segregation have been reported suggesting existence of other yet unknown genetic factors contributing to observed phenotypes. Thus, authors emphasize the need for caution during genetic counseling for families with *MYH11* mutations and patients not carrying family-specific *MYH11* variant should not be excluded from further surveillance [44]. This might also be the case in the family TAAD103 in which p.E1840D proband’s granddaughter was diagnosed with PDA while her mother was negatively tested for p.E1840D. However in many cases there is not enough data to determine if *MYH11* variants (including p.E1840D and p.R1758Q) are sufficient to cause TAA or should be classified as VUS. However, considering common clinical traits (in our unrelated patients we found lack of PDA and the presence of bicuspid aortic valve, Figs. 5, 6) and relative proximity of both variants within coiled coil domain, we decided to classify them as likely pathogenic.

### ACTA2

Both *ACTA2* mutations detected in our cohort (p.D27G and p.N117S) are novel, although another change at residue 117 as well as adjacent 118 have been reported in families with *ACTA2* associated vascular diseases including TAAD and premature CAD or stroke [45].

*ACTA2* p.D27G was found in a 41 years old patient (family TAAD111), hypertensive, following AAD at the age of 29 years, operated with insertion of supracoronary aortic prosthesis 29 mm, with chronic dissection of thoraco-abdominal aorta and positive family history for aggressive TAAD. Both his father and father’s sister suffered from AAD and died during the corrective surgery at the age of 44 and 52 years, respectively. Their mother had planned surgery because of TAA at the age of 70 years. The patient did not exhibit any features of any other tissue involvement, no iris flocculi. His son is a 19 years old asymptomatic mutation carrier.

*ACTA2* p.N117S was detected in a 29-year-old female (family TAAD076) who suffered type B AAD at the age of 18 while playing tennis. Within a decade she required three farther corrective procedures on her thoraco-abdominal aorta. She does not present any other tissue involvement. At the age of 29 years she had also TAA with Z-score 2.7. The proband’s mother is an asymptomatic mutation carrier. She was diagnosed for mild arterial hypertension and hypercholesterolemia, but transthoracic echocardiography revealed normal aortic dimensions. No iris flocculi were found in the mutation carriers. The proband’s uncle, also mutation carrier had premature ischemic stroke at the age of 39 years and premature myocardial infarction at the age of 42. During the following years, he required repeated percutaneous coronary interventions and finally, at the age of 50 he underwent coronary artery by-pass grafting. On available medical records his aorta was within normal range.

### COL3A1

Over 95 % of pathogenic missense variants in *COL3A1* are substitutions of glycine–amino acid essential for the assembly of collagen triple helix. Remaining missense variants are reported as benign or VUS. Same applies to p.P703L which in absence of any pedigree-derived evidence indicating its pathogenicity must be classified as VUS. p.I1290T however is located in noncollagenous C-terminal globular (NC1) domain responsible for both fibril formation and cross-linking between collagen molecules in the extracellular matrix [46]. At least one other variant in this region has been reported in EDS type IV [47]. Combining this data with segregation analysis and software predictions, we classified this variant as likely pathogenic. p.I1290T was found in a 55 years old proband (family TAAD097), who suffered from Stanford type A AAD at age of 40 years, and was re-operated at the age of 51 because of progressive aortic root dilatation with severe aortic insufficiency along with coronary artery bypass grafting (LIMA-LAD) due to CAD. His sister was diagnosed with TAA (aortic root Z-score 3.4), and his son at age of 33 years met borderline criteria for the diagnosis of TAA. The proband’s daughter is the mutation carrier with normal aortic dimensions.

### MYLK

Two (p.R378H and p.T690M) out of three rare missense variants we have found in the *MYLK* gene showed no segregation with the disease and we observed no modifying effect on patients’ phenotypes so we classified them as likely benign. The third *MYLK* variant (p.P203L) was considered as VUS after considering both ambiguous results given by pathogenicity estimating software and lack of definite segregation.

## Conclusions

This study broadens the spectrum of genetic background of thoracic aneurysms and dissections and supports its potential role as a prognostic factor in the patients with the disease.

## Additional file

10.1186/s12967-016-0870-4 Complementary data on primer sequences, SKI amplification and survival analyses.

## Abbreviations

AAD: acute aortic dissections
ASD: atrial septal defect
BAV: bicuspid aortic valve
CABG: coronary artery bypass graft
CAD: coronary artery disease
CNV: copy number variation
CoA: coarctation of the aorta
EDS: Ehlers-Danlos syndrome
FTAAD: familial thoracic aortic aneurysms and dissections
LDS: Loyes-Dietz syndrome
LVEDD: left ventricular end diastolic diameter
LVEF: left ventricular ejection fraction
LVNC: left ventricular noncompaction
MFS: Marfan syndrome
MI: myocardial infarction
MR: mitral regurgitation
MVP: mitral valve prolapse
NGS: next generation sequencing
PCI: prophylactic cranial irradiation
PDA: patent ductus arteriosus
TAA: thoracic aortic aneurysms
TAAD: thoracic aortic aneurysms and dissections
TSO: TruSight One
VUS: variant of unknown significance
WES: whole exome sequencing

## References

[1] Hiratzka LF, Bakris GL, Beckman JA, Bersin RM, Carr VF, Casey DE (2010). ACCF/AHA/AATS/ACR/ASA/SCA/SCAI/SIR/STS/SVM guidelines for the diagnosis and management of patients with thoracic aortic disease: a report of the American College of Cardiology Foundation/American Heart Association Task Force on Practice Guidelines, American Association for Thoracic Surgery, American College of Radiology, American Stroke Association, Society of Cardiovascular Anesthesiologists, Society for Cardiovascular Angiography and Interventions, Society of Interventional Radiology, Society of Thoracic Surgeons, and Society for Vascular Medicine. Circulation.

[2] Elefteriades JA, Farkas EA (2010). Thoracic aortic aneurysm clinically pertinent controversies and uncertainties. J Am Coll Cardiol.

[3] Albornoz G, Coady MA, Roberts M, Davies RR, Tranquilli M, Rizzo JA (2006). Familial thoracic aortic aneurysms and dissections–incidence, modes of inheritance, and phenotypic patterns. Ann Thorac Surg.

[4] Disabella E, Grasso M, Gambarin FI, Narula N, Dore R, Favalli V (2011). Risk of dissection in thoracic aneurysms associated with mutations of smooth muscle alpha-actin 2 (ACTA2). Heart.

[5] Milewicz DM, Carlson AA, Regalado ES (2010). Genetic testing in aortic aneurysm disease: PRO. Cardiol Clin.

[6] Lee B, Godfrey M, Vitale E, Hori H, Mattei MG, Sarfarazi M (1991). Linkage of Marfan syndrome and a phenotypically related disorder to two different fibrillin genes. Nature.

[7] Maslen CL, Corson GM, Maddox BK, Glanville RW, Sakai LY (1991). Partial sequence of a candidate gene for the Marfan syndrome. Nature.

[8] Dietz HC, Cutting GR, Pyeritz RE, Maslen CL, Sakai LY, Corson GM (1991). Marfan syndrome caused by a recurrent de novo missense mutation in the fibrillin gene. Nature.

[9] Kroes HY, Pals G, van Essen AJ (2003). Ehlers-Danlos syndrome type IV: unusual congenital anomalies in a mother and son with a COL3A1 mutation and a normal collagen III protein profile. Clin Genet.

[10] Mizuguchi T, Collod-Beroud G, Akiyama T, Abifadel M, Harada N, Morisaki T (2004). Heterozygous TGFBR2 mutations in Marfan syndrome. Nat Genet.

[11] Loeys BL, Chen J, Neptune ER, Judge DP, Podowski M, Holm T (2005). A syndrome of altered cardiovascular, craniofacial, neurocognitive and skeletal development caused by mutations in TGFBR1 or TGFBR2. Nat Genet.

[12] Zhu L, Vranckx R, Khau Van Kien P, Lalande A, Boisset N, Mathieu F (2006). Mutations in myosin heavy chain 11 cause a syndrome associating thoracic aortic aneurysm/aortic dissection and patent ductus arteriosus. Nat Genet.

[13] Guo DC, Pannu H, Tran-Fadulu V, Papke CL, Yu RK, Avidan N (2007). Mutations in smooth muscle alpha-actin (ACTA2) lead to thoracic aortic aneurysms and dissections. Nat Genet.

[14] Loeys BL, Dietz HC, Braverman AC, Callewaert BL, De Backer J, Devereux RB (2010). The revised Ghent nosology for the Marfan syndrome. J Med Genet.

[15] Greally MT. Shprintzen–Goldberg Syndrome. 1993. doi: NBK1277 [bookaccession]. 20301454

[16] Freed LA, Benjamin EJ, Levy D, Larson MG, Evans JC, Fuller DL (2002). Mitral valve prolapse in the general population: the benign nature of echocardiographic features in the Framingham Heart Study. J Am Coll Cardiol.

[17] Ploski R, Pollak A, Muller S, Franaszczyk M, Michalak E, Kosinska J (2014). Does p. Q247X in TRIM63 cause human hypertrophic cardiomyopathy?. Circ Res.

[18] Naz S, Fatima A (2013). Amplification of GC-rich DNA for high-throughput family-based genetic studies. Mol Biotechnol.

[19] Wooderchak-Donahue W, VanSant-Webb C, Tvrdik T, Plant P, Lewis T, Stocks J (2015). Clinical utility of a next generation sequencing panel assay for Marfan and Marfan-like syndromes featuring aortopathy. Am J Med Genet A..

[20] Proost D, Vandeweyer G, Meester JA, Salemink S, Kempers M, Ingram C (2015). Performant mutation identification using targeted next-generation sequencing of 14 thoracic aortic aneurysm genes. Hum Mutat.

[21] Ziganshin BA, Bailey AE, Coons C, Dykas D, Charilaou P, Tanriverdi LH (2015). Routine genetic testing for thoracic aortic aneurysm and dissection in a clinical setting. Ann Thorac Surg.

[22] Tiecke F, Katzke S, Booms P, Robinson PN, Neumann L, Godfrey M (2001). Classic, atypically severe and neonatal Marfan syndrome: twelve mutations and genotype-phenotype correlations in FBN1 exons 24-40. Eur J Hum Genet.

[23] Yoo EH, Woo H, Ki CS, Lee HJ, Kim DK, Kang IS (2010). Clinical and genetic analysis of Korean patients with Marfan syndrome: possible ethnic differences in clinical manifestation. Clin Genet.

[24] Stheneur C, Collod-Beroud G, Faivre L, Buyck JF, Gouya L, Le Parc JM (2009). Identification of the minimal combination of clinical features in probands for efficient mutation detection in the FBN1 gene. Eur J Hum Genet.

[25] Aalberts JJ, van Tintelen JP, Meijboom LJ, Polko A, Jongbloed JD, van der Wal H (2014). Relation between genotype and left-ventricular dilatation in patients with Marfan syndrome. Gene.

[26] Ogawa N, Imai Y, Takahashi Y, Nawata K, Hara K, Nishimura H (2011). Evaluating Japanese patients with the Marfan syndrome using high-throughput microarray-based mutational analysis of fibrillin-1 gene. Am J Cardiol.

[27] Loeys B, Nuytinck L, Delvaux I, De Bie S, De Paepe A (2001). Genotype and phenotype analysis of 171 patients referred for molecular study of the fibrillin-1 gene FBN1 because of suspected Marfan syndrome. Arch Intern Med.

[28] Collod-Beroud G, Le Bourdelles S, Ades L, Ala-Kokko L, Booms P, Boxer M (2003). Update of the UMD-FBN1 mutation database and creation of an FBN1 polymorphism database. Hum Mutat.

[29] Comeglio P, Johnson P, Arno G, Brice G, Evans A, Aragon-Martin J (2007). The importance of mutation detection in Marfan syndrome and Marfan-related disorders: report of 193 FBN1 mutations. Hum Mutat.

[30] Howarth R, Yearwood C, Harvey JF (2007). Application of dHPLC for mutation detection of the fibrillin-1 gene for the diagnosis of Marfan syndrome in a National Health Service Laboratory. Genet Test..

[31] Soylen B, Singh KK, Abuzainin A, Rommel K, Becker H, Arslan-Kirchner M (2009). Prevalence of dural ectasia in 63 gene-mutation-positive patients with features of Marfan syndrome type 1 and Loeys-Dietz syndrome and report of 22 novel FBN1 mutations. Clin Genet.

[32] Waldmuller S, Muller M, Warnecke H, Rees W, Schols W, Walterbusch G (2007). Genetic testing in patients with aortic aneurysms/dissections: a novel genotype/phenotype correlation?. Eur J Cardiothorac Surg.

[33] Faivre L, Gorlin RJ, Wirtz MK, Godfrey M, Dagoneau N, Samples JR (2003). In frame fibrillin-1 gene deletion in autosomal dominant Weill-Marchesani syndrome. J Med Genet.

[34] Matyas G, De Paepe A, Halliday D, Boileau C, Pals G, Steinmann B (2002). Evaluation and application of denaturing HPLC for mutation detection in Marfan syndrome: identification of 20 novel mutations and two novel polymorphisms in the FBN1 gene. Hum Mutat.

[35] Robinson DO, Lin F, Lyon M, Raponi M, Cross E, White HE (2012). Systematic screening of FBN1 gene unclassified missense variants for splice abnormalities. Clin Genet.

[36] Attanasio M, Pratelli E, Porciani MC, Evangelisti L, Torricelli E, Pellicano G (2013). Dural ectasia and FBN1 mutation screening of 40 patients with Marfan syndrome and related disorders: role of dural ectasia for the diagnosis. Eur J Med Genet..

[37] Aalberts JJ, Schuurman AG, Pals G, Hamel BJ, Bosman G, Hilhorst-Hofstee Y (2010). Recurrent and founder mutations in the Netherlands: extensive clinical variability in Marfan syndrome patients with a single novel recurrent fibrillin-1 missense mutation. Neth Heart J..

[38] Grau U, Klein HG, Detter C, Mair H, Welz A, Seidel D (1998). A novel mutation in the neonatal region of the fibrillin (FBN)1 gene associated with a classical phenotype of Marfan syndrome (MfS). Mutations in brief no. 163. Online. Hum Mutat.

[39] Arslan-Kirchner M, Epplen JT, Faivre L, Jondeau G, Schmidtke J, De Paepe A et al. Clinical utility gene card for: Loeys-Dietz syndrome (TGFBR1/2) and related phenotypes. Eur J Hum Genet. 2011;19(10). doi:10.1038/ejhg.2011.68. 10.1038/ejhg.2011.68 PMC3190257 21522183

[40] Frischmeyer-Guerrerio PA, Guerrerio AL, Oswald G, Chichester K, Myers L, Halushka MK et al. TGFbeta receptor mutations impose a strong predisposition for human allergic disease. Sci Transl Med. 2013;5(195):195ra94. doi:10.1126/scitranslmed.3006448. 10.1126/scitranslmed.3006448 PMC3905327 23884466

[41] Schepers D, Doyle AJ, Oswald G, Sparks E, Myers L, Willems PJ (2015). The SMAD-binding domain of SKI: a hotspot for de novo mutations causing Shprintzen-Goldberg syndrome. Eur J Hum Genet.

[42] van de Laar IM, Oldenburg RA, Pals G, Roos-Hesselink JW, de Graaf BM, Verhagen JM (2011). Mutations in SMAD3 cause a syndromic form of aortic aneurysms and dissections with early-onset osteoarthritis. Nat Genet.

[43] van de Laar IM, van der Linde D, Oei EH, Bos PK, Bessems JH, Bierma-Zeinstra SM (2012). Phenotypic spectrum of the SMAD3-related aneurysms-osteoarthritis syndrome. J Med Genet.

[44] Harakalova M, van der Smagt J, de Kovel CG, Van’t Slot R, Poot M, Nijman IJ (2013). Incomplete segregation of MYH11 variants with thoracic aortic aneurysms and dissections and patent ductus arteriosus. Eur J Hum Genet.

[45] Guo DC, Papke CL, Tran-Fadulu V, Regalado ES, Avidan N, Johnson RJ (2009). Mutations in smooth muscle alpha-actin (ACTA2) cause coronary artery disease, stroke, and Moyamoya disease, along with thoracic aortic disease. Am J Hum Genet.

[46] Henkel W (1996). Cross-link analysis of the C-telopeptide domain from type III collagen. Biochem J.

[47] Morissette R, Schoenhoff F, Xu Z, Shilane DA, Griswold BF, Chen W (2014). Transforming growth factor-beta and inflammation in vascular (type IV) Ehlers-Danlos syndrome. Circ Cardiovasc Genet..

[48] Liu WO, Oefner PJ, Qian C, Odom RS, Francke U (1997). Denaturing HPLC-identified novel FBN1 mutations, polymorphisms, and sequence variants in Marfan syndrome and related connective tissue disorders. Genet Test..

