# Supplementary material for: Next-generation sequencing for diagnosis of thoracic aortic aneurysms and dissections: diagnostic yield, novel mutations and genotype phenotype correlations
Source: J Transl Med. 2016 May 4;14:115. doi: 10.1186/s12967-016-0870-4 (PMC4855821; doi:10.1186/s12967-016-0870-4)

| **No** | **Gene** | **Variant** | **Forward primer** | **Reversed primer** |
| --- | --- | --- | --- | --- |
| **1** | ***FBN1*** | **c.6740-2A>G** | CAGCCAGTAGTGAAATAACA | CACCATGGAGAGTCCTGACA |
| **2** | ***FBN1*** | **N2502X** | AGCCACCTCTGCCTGTCTTA | GATTACAAAAAGCATGGTTCTCC |
| **3** | ***FBN1*** | **C1408F** | AACATTGCTGCACTGGAAAG | AAAAAGCATCAGGAATGTTTA |
| **4** | ***FBN1*** | **I2585T** | AGAATAATGTGTAGGATGTGT | CAGAAAGCAAGCAGTGTTTT |
| **5** | ***FBN1*** | **R1692del** | AATGACTTAGTTCTGTGGCATGA | CTGATTTCCCCAACAATTCA |
| **6** | ***FBN1*** | **Y2639C** | AGCCACCTCTGCCTGTCTTA | GATTACAAAAAGCATGGTTCTCC |
| **7** | ***FBN1*** | **G744E** | GGAACTTGTGGGAGAAAGATTGG | CAAAGGCAGTTTTCTCCCAGC |
| **8** | ***FBN1*** | **V984I** | CAGTTTGGGGCAGTGGAAG | TGATCAAGTAGAGTGCTGAGATCAT |
| **9** | ***TGFBR1*** | **A202V** | ACAAAGCCTACCTTGACCCTC | TGGGTCTAATCTACATGAGAGACA |
| **10** | ***TGFBR1*** | **Y282H** | GGTGGCATTATATTGCAGTGT | TTATAGGCATGAGCCACCAG |
| **11** | ***TGFBR2*** | **A527T** | GGTGCCCTTTGGATCTCTTT | CCGAGAGCCTGTCCAGATG |
| **12** | ***SKI*** | **T20K** | GCGGCGCGGGGCGCGTGGATGT | GCGGCGGCTGGATGGCGGGCAGGT |
| **13** | ***SMAD3*** | **I290F** | GGGGAAATGGTTTTCCAGAG | CCTTCAGAGGCTGTGTGTTC |
| **14** | ***ACTA2*** | **D27G** | CCACAAATGCCCAATTACAG | AACTTCTGGGCAGAAAGAGAT |
| **15** | ***ACTA2*** | **N117S** | GGGAAATGCAGGACCTCAT | ACAGAAGTTCCCCAGACCC |
| **16** | ***MYH11*** | **R1758Q** | CTCCAAGCCTCAGTTTCACC | ACCCTCTTGTCCCTCAATCC |
| **17** | ***MYH11*** | **E1833D** | AAGAGGCATGGTCTCAGTGC | TGGAAGAGGTTCCCTGACTT |
| **18** | ***COL3A1*** | **P703L** | TGACAGCTCACACTTAACCAGA | TTGTTGCTTTAGGAATCCATCTTAAA |
| **19** | ***COL3A1*** | **I1290T** | TCAACATTATGAATGCCTTTACAGGT | ACCAACCTAGTAACTTTGCTTTGTG |
| **20** | ***MYLK*** | **P203L** | AGGAGGCAGGAGGACACAG | GCAGTGAACAAGCAGCTCCT |
| **21** | ***MYLK*** | **R378H** | GCCAAAAGCAAGAACTGCTC | GACTAGCTGGGTGCTGAAGG |
| **22** | ***MYLK*** | **T690M** | ATCTGGCTGCACAATGGGAA | CAGCCTCACCTTGTACCGTG |

**Supplementary table 1**. Primer pairs used for Sanger sequencing

**SKI ex 1a amplification and sequencing**

PCR reaction mix (20μl):

3.75μl H2O,

1.5 dNTPs (1,3 mM),

1.3μl Mg_2_Cl (25mM),

3.5μl forward primer (10 mM),

3.5μl reversed primer (10 mM),

4μl 5X Green GoTaq Flexi Reaction Buffer,

0.25μl Taq polymerase (5U/μl, Thermo Scientific),

0.25μl formamide,

1.2μl DMSO,

0,25 μl β-mercaptoethanol (99,9%)

Amplification:

94C - 5’, (94°C - 0:45’,70°C - 0:20’, 72°C - 1’)x40, 10’- 72°C

Sequencing PRC reaction mix (10μl):

2μl BigDye® Terminator v3.1 Cycle Sequencing Kit,

2μl buffer, 3μl primer (1pM),

1μl DMSO,

2μl cleaned PCR product

**Supplementary figure 1**. Event free survival among TAAD probands with variants classified as likely causative vs. those without any candidate variants identified (Log Rank Chi-square 6.79, P=0.0092).


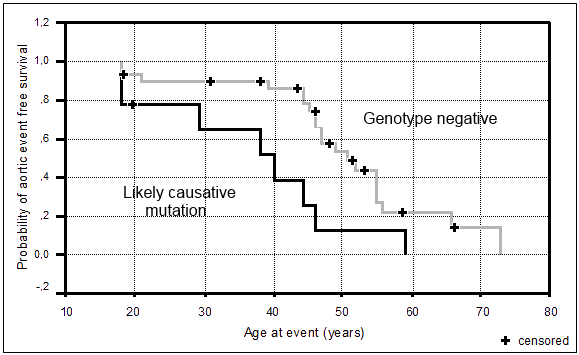


**Supplementary figure 2**. Event free survival among TAAD probands with variants in genes involved in TGF beta signaling vs. those without any candidate variants identified (Log Rank Chi-square 8.66, P=0.0033).

.
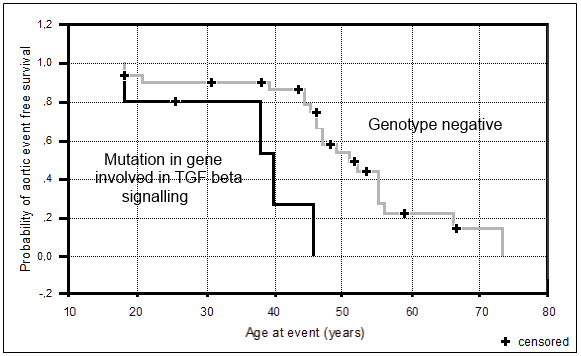

Supplement: Supplementary file 1 — 10.1186/s12967-016-0870-4 Complementary data on primer sequences, SKI amplification and survival analyses. [file 12967_2016_870_MOESM1_ESM.docx]
